# Differential physiological roles for BIN1 isoforms in skeletal muscle development, function and regeneration

**DOI:** 10.1242/dmm.044354

**Published:** 2020-11-24

**Authors:** Ivana Prokic, Belinda S. Cowling, Candice Kutchukian, Christine Kretz, Hichem Tasfaout, Vincent Gache, Josiane Hergueux, Olivia Wendling, Arnaud Ferry, Anne Toussaint, Christos Gavriilidis, Vasugi Nattarayan, Catherine Koch, Jeanne Lainé, Roy Combe, Laurent Tiret, Vincent Jacquemond, Fanny Pilot-Storck, Jocelyn Laporte

**Affiliations:** 1 Institut de Génétique et de Biologie Moléculaire et Cellulaire (IGBMC), Department of Translational Medicine, 67404 Illkirch, France; 2 Centre National de la Recherche Scientifique (CNRS), UMR7104, 67404 Illkirch, France; 3 Institut National de la Santé et de la Recherche Médicale (INSERM), U1258, 67404 Illkirch, France; 4 Université de Strasbourg, 67404 Illkirch, France; 5 Université Lyon, Université Claude Bernard Lyon 1, CNRS UMR-5310, INSERM U-1217, Institut NeuroMyoGène, 8 Avenue Rockefeller, 69373 Lyon, France; 6 Sorbonne Université, INSERM, Institute of Myology, Centre of Research in Myology, Unité Mixte de Recherche (UMRS) 794, 75013 Paris, France; 7 Sorbonne Université, INSERM, Institute of Myology, Centre of Research in Myology, Department of Physiology, UMRS 974, 75013 Paris, France; 8 Sorbonne Université, Department of Physiology, Université Paris 06, Pitié-Salpêtrière Hospital, 75013 Paris, France; 9 CELPHEDIA-PHENOMIN, Institut Clinique de la Souris (ICS), 67404 Illkirch, France; 10 Université Paris Est Creteil, INSERM, EnvA, EFS, AP-HP, IMRB, BNMS Team, 94700 Maisons-Alfort, France

**Keywords:** Myotubular myopathy, Centronuclear myopathy, XLMTM, SH3 domain, BAR domain, Myotonic dystrophy, Triad, Dynamin, Myoblast fusion, Animal model

## Abstract

Skeletal muscle development and regeneration are tightly regulated processes. How the intracellular organization of muscle fibers is achieved during these steps is unclear. Here, we focus on the cellular and physiological roles of amphiphysin 2 (BIN1), a membrane remodeling protein mutated in both congenital and adult centronuclear myopathies (CNM), that is ubiquitously expressed and has skeletal muscle-specific isoforms. We created and characterized constitutive muscle-specific and inducible *Bin1* homozygous and heterozygous knockout mice targeting either ubiquitous or muscle-specific isoforms. Constitutive *Bin1*-deficient mice died at birth from lack of feeding due to a skeletal muscle defect. T-tubules and other organelles were misplaced and altered, supporting a general early role for BIN1 in intracellular organization, in addition to membrane remodeling. Although restricted deletion of *Bin1* in unchallenged adult muscles had no impact, the forced switch from the muscle-specific isoforms to the ubiquitous isoforms through deletion of the in-frame muscle-specific exon delayed muscle regeneration. Thus, ubiquitous BIN1 function is necessary for muscle development and function, whereas its muscle-specific isoforms fine tune muscle regeneration in adulthood, supporting that BIN1 CNM with congenital onset are due to developmental defects, whereas later onset may be due to regeneration defects.

## Abstract (precis)

**Summary:** The *BIN1* gene, mutated in centronuclear myopathies, expresses ubiquitous and muscle-specific isoforms. It is shown here that ubiquitous isoforms are necessary for muscle development, whereas muscle-specific isoforms fine-tune muscle regeneration.

## INTRODUCTION

Skeletal muscle is composed of bundles of multinucleated myofibers containing dozens to thousands of nuclei in a common cytoplasm. Specific organelles, such as the myofibril contractile apparatus and the triads, sustain contraction and force development (Franzini-Armstrong, 2018). The triad sustains the excitation-contraction (EC) coupling machinery and is composed of a plasma membrane invagination (T-tubule) connecting two terminal cisternae from the sarcoplasmic reticulum (SR). The membrane network is exquisitely developed in muscle with the SR wrapping the myofibrils (Towler et al., 2004). During muscle development in mammals, other organelles achieve specific localization: e.g. mitochondria concentrate around myofibrils, whereas nuclei are positioned at the periphery of the fiber. Underneath the basal lamina that surrounds each myofiber are satellite cells (Mauro, 1961), which are the muscle stem cells implicated in muscle growth and regeneration. In mammals, several steps of muscle remodeling occur: muscle development before birth; maturation around and shortly after birth; muscle maintenance; and muscle regeneration upon injury (Engel and Franzini-Armstrong, 2004). Muscle regeneration recapitulates to a certain extent the developmental process of muscle formation. Satellite cells proliferate in response to growth factors produced following muscle injury and fuse with existing myofibers. A plethora of muscle diseases impair different steps of muscle formation or maintenance (Demonbreun and McNally, 2016). Hence, understanding the key players in these processes represents a crucial step towards the development of therapeutic approaches. Here, we investigated the physiological role of BIN1, a protein mutated in rare myopathies, on muscle development, function and regeneration.

BIN1, or amphiphysin 2 (also known as bridging integrator 1), is a main regulator of membrane remodeling and trafficking (McMahon and Gallop, 2005; Itoh and De Camilli, 2006; Prokic et al., 2014), and its role in organelle positioning has begun to emerge (Falcone et al., 2014; D'Alessandro et al., 2015). It belongs to the amphiphysin protein family, in which the N-terminal BAR domain is known to recognize and induce membrane curvature (Peter et al., 2004; Frost et al., 2009). It also has an SH3 domain binding to proline-rich motifs in other proteins. BIN1 is a ubiquitously expressed protein that is highly expressed in skeletal muscles and the brain (Sakamuro et al., 1996; Butler et al., 1997). The *BIN1* gene has 20 exons and encodes multiple isoforms (Table S1) (Prokic et al., 2014). Skeletal muscle expresses several isoforms including ubiquitous isoforms that contain both the BAR and C-terminal SH3 domains and muscle-specific isoforms that in addition contain the in-frame exon 11 (named exon 10 in previous studies on the cDNA) encoding a polybasic motif binding phosphoinositides (PIs) (Lee et al., 2002; Fugier et al., 2011). The PI^+^ muscle-specific isoforms appear during muscle development and are the prevalent isoforms in adulthood (Cowling et al., 2017). Overexpression of the muscle-specific BIN1 iso8 isoform (a PI^+^ isoform) but not the ubiquitous BIN1 iso9 (PI^−^) isoform in C2C12 myoblasts and other cells results in the formation of numerous tubules connected to the plasma membrane, suggesting the PI domain promotes membrane remodeling (Lee et al., 2002; Nicot et al., 2007; Böhm et al., 2013). In addition to binding phosphoinositides, the muscle-specific PI motif regulates a conformational switch by binding the SH3 domain of BIN1 molecules (Kojima et al., 2004; Royer et al., 2013). The abundance of PIs, such as PtdIns(4,5)P_2_, may regulate this conformational switch and therefore the binding to downstream interactors of the SH3 domain, such as dynamin or synaptojanin (Kojima et al., 2004).

*BIN1* is mutated in different forms of centronuclear myopathies (CNM), characterized by muscle hypotrophy, muscle weakness, centralized nuclei and triad defects (Romero, 2010; Toussaint et al., 2011; Jungbluth and Gautel, 2014; Prokic et al., 2014). Considering recessive CNM cases with non-progressive muscle involvement, homozygous missense mutations in the BAR domain impair the membrane tubulation properties, and premature truncation of the SH3 domain reduces the binding to dynamin 2 (DNM2) (Nicot et al., 2007). Moreover, a splice site mutation leading to the skipping of in-frame exon 11 was identified in humans and dogs presenting with highly progressive CNM (Böhm et al., 2013). Exon 11 skipping was also associated with myotonic dystrophies (DM), characterized by muscle wasting and increased fiber degeneration and regeneration (Fugier et al., 2011). In addition, heterozygous BIN1 mutations cause an adult-onset mild CNM (Böhm et al., 2014). In all cases, the primary affected organ is the skeletal muscle but how *BIN1* mutations in different domains lead to different onset and severity is not understood. Findings in cells and animal models suggested a role for BIN1 in the formation of T-tubules (Razzaq et al., 2001; Lee et al., 2002; Tjondrokoesoemo et al., 2011; Böhm et al., 2013; Smith et al., 2014). However, the physiological role of BIN1 in mammalian muscle remains elusive.

In this study, we characterized several novel *Bin1* knockout (KO) mice (Table S2). In *Bin1* KO mice deleted for exon 20 (*Bin1*ex20^−/−^), the SH3 domain in all isoforms was disrupted, similarly as in some CNM patients with truncating mutations, which makes these mice a good model to study the ubiquitous function of BIN1. In *Bin1* KO mice missing exon 11 (*Bin1*ex11^−/−^), the muscle-specific isoforms were converted into ubiquitous isoforms by deletion of this in-frame exon. This later model helped address the muscle-specific function of PI^+^ BIN1 isoforms. Molecular, cellular and physiological characterization of constitutive, muscle-specific and inducible lines uncovered that although BIN1 is necessary for skeletal muscle development and function at birth, its muscle-specific isoforms are dispensable for development but required for muscle regeneration at adulthood.

## RESULTS

### *Bin1* has the highest expression in skeletal muscle, in which it is mainly located on the triads

**Fig. 1.**
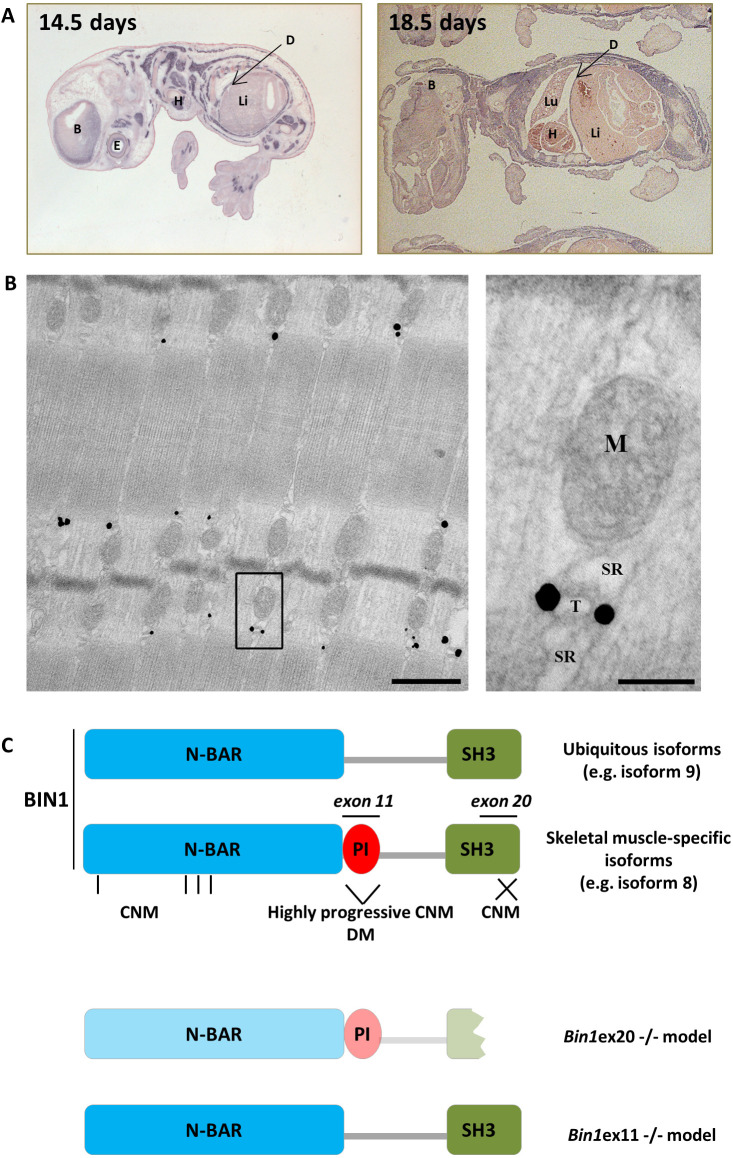
**BIN1 expression and localization.** (A) *In situ Bin1* labeling in wild-type mouse at E14.5 and E18.5. (B) Immunogold labeling using anti-PI domain-specific antibody on a TA muscle from a wild-type adult mouse showing specific triad localization. Scale bars: 500 nm (left panel); 100 nm (right panel). (C) BIN1 functional domains encompass the N-BAR (N-terminal amphipathic helix with Bin-Amph-Rvs sequences), the PI (phosphoinositides binding), and the SH3 (src homology) domains. Exon 11 encodes the muscle-specific PI domain, whereas exon 20 encodes the second half of the ubiquitous SH3 domain. Missense mutations in the N-BAR (vertical bars) or truncation of the SH3 domains (cross) lead to recessive CNM, whereas skipping of the PI domain is linked to highly progressive CNM and DM. Bottom: *Bin1*ex20^−/−^ mice express a strongly reduced and truncated BIN1, whereas *Bin1*ex11^−/−^ mice express the ubiquitous isoforms in muscle. B, brain; D, diaphragm; E, eye; H, heart; Li, liver; Lu, lung, M, mitochondria; SR, sarcoplasmic reticulum; T, T-tubule.

In adult humans, *BIN1* is ubiquitously expressed with the highest expression in skeletal muscle (Fig. S1A) (Sakamuro et al., 1996; Butler et al., 1997; Nicot et al., 2007; GTEx consortium, 2015). To address *Bin1* expression during mouse embryonic development we performed *in situ* hybridization at embryonic (E) day 14.5 and E18.5 using a probe against the 3′UTR region of *Bin1* (Fig. 1A). The highest expression of *Bin1* was found in skeletal muscle and the diaphragm, followed by the cortex and the eye. To assess the localization of muscle-specific BIN1 isoforms in skeletal muscle, immunogold labeling was performed in the adult tibialis anterior (TA) muscle using an anti-BIN1 antibody directed against the PI domain encoded by the muscle-specific exon 11. This staining revealed that muscle-specific BIN1 localizes at the triads but not to the SR, mitochondria or the myofilaments (Fig. 1B). These findings suggested an important role for BIN1 in muscle development for the formation of triads.

### Constitutive and muscle-specific homozygous deletions of *Bin1* exon 20 lead to feeding inability and perinatal death

To address the physiological importance of BIN1 and generate a BIN1-CNM model, we created and analyzed *Bin1*ex20^−/−^ mice, in which the last exon, 20, is deleted (Fig. 1C), thus causing disruption of the SH3 domain similar to some CNM patients (Fig. S1B,C) (Cowling et al., 2017). The constitutive homozygous deletion of *Bin1* is lethal in the first hours after birth and cardiomyopathy was proposed to be the fatal cause (Muller et al., 2003). However, mice with a cardiac-specific deletion of *Bin1* survived (Laury-Kleintop et al., 2015). To further clarify the death-causing mechanism of *Bin1* deficiency, we analyzed survival. We confirmed that this constitutive deletion induced precocious lethality, with no *Bin1*ex20^−/−^ mice surviving the first postnatal day among 131 littermates. Homozygous deletion of *Bin1* exon 20 led to a strong decrease in the protein level, and there was no compensatory overexpression of its close homolog amphiphysin 1 (Fig. S1D,E). Comparison of *Bin1*ex20^−/−^ and wild-type littermates showed no differences in body weight or in heart organization and functioning (Fig. S2A-E).

**Fig. 2.**
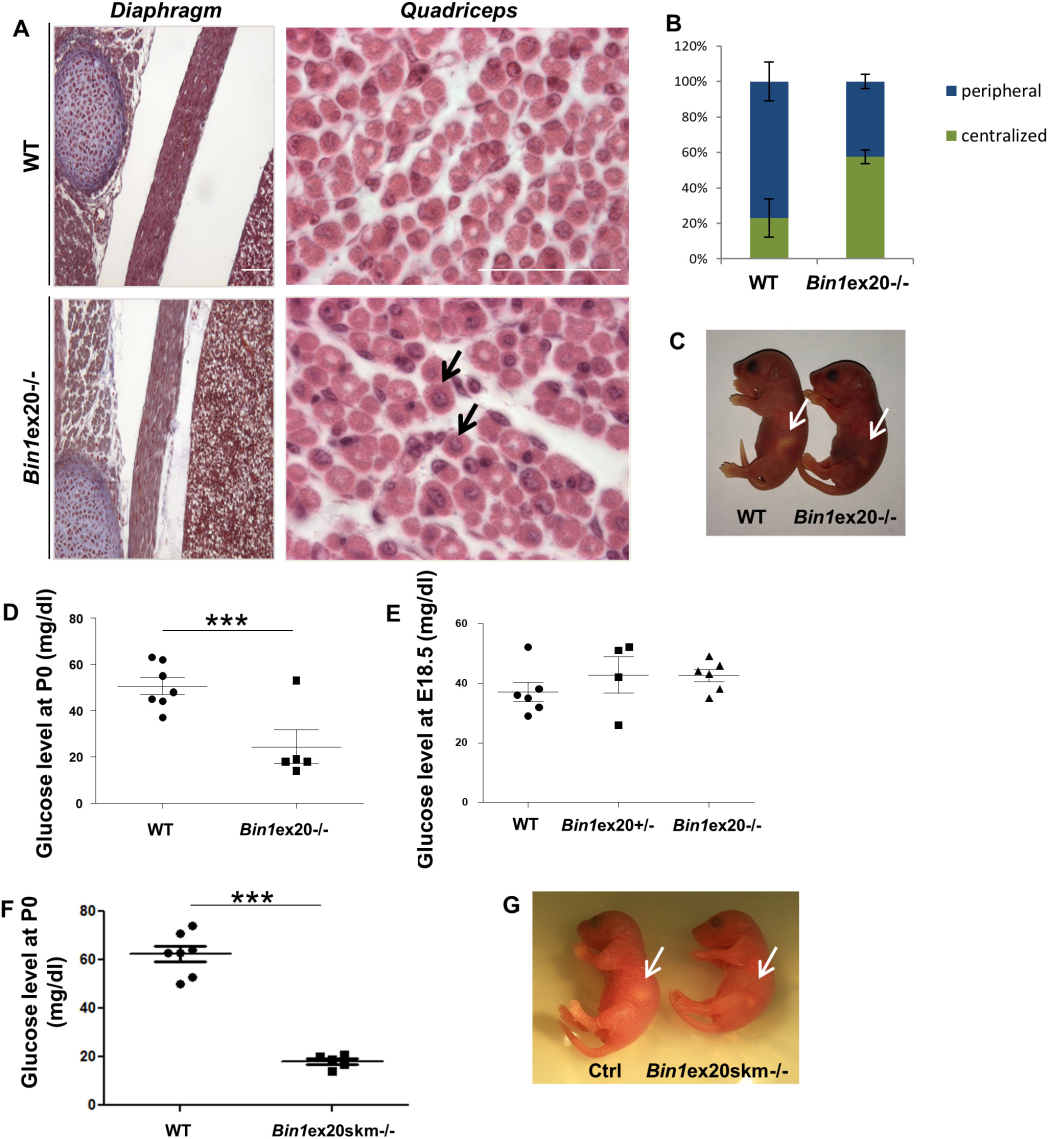
**Muscle-related lethality in *Bin1*ex20^−/−^** **mice.** (A) Histology of the diaphragm and quadriceps in newborn wild-type (WT) and *Bin1*ex20^−/−^ mice showing centralized nuclei in the quadriceps (arrows). Scale bars: 200 µm. (B) Quantification of centralized nuclei versus peripheral nuclei in quadriceps. (C) *Bin1*ex20^−/−^ mice had an empty stomach (arrows) compared to wild-type littermates. (D) Blood glucose level in P0 newborns. Most *Bin1*ex20^−/−^ mice showed a threefold reduction in glucose level compared to wild-type littermates (*n*≥5 mice per group). (E) Blood glucose level in E18.5 embryos showing no difference in the glucose level between the wild-type, *Bin1*ex20^+/−^ and constitutive *Bin1*ex20^−/−^ mice (*n*≥4 mice per group). (F) Glucose measurement at P0 in muscle-specific *Bin1*ex20skm^−/−^ mice showed a threefold reduction compared to wild-type littermates. (G) *Bin1*ex20skm^−/−^ mice had an empty stomach (arrows) compared to wild-type littermates. (*n*≥5 mice per group). Unpaired Student's *t*-test. Data are mean±s.e.m. ***P<0.001.

We then used the Cre recombinase under the control of the human skeletal actin promoter to induce the deletion specifically in skeletal muscle. As in the case of constitutive KO, the muscle-specific homozygous deletion led to a strong BIN1 reduction, with no *Bin1*ex20skm^−/−^ mice surviving the first hours after birth (Fig. 2; Fig. S1F). A muscle-based lethality could originate either from breathing or a feeding defect. Hematoxylin and Eosin (H&E) staining of the diaphragm from *Bin1*ex20^−/−^ mice showed that organization and thickness did not differ from control mice (Fig. 2A). Accordingly, their lungs inflated, and no cyanosis was observed, suggesting a functional diaphragm. Conversely, H&E analysis of the quadriceps of constitutive *Bin1*ex20^−/−^ mice revealed increased centralization of nuclei, a hallmark of CNM (Fig. 2A,B). Even several hours after birth and unlike their wild-type littermates, constitutive *Bin1*ex20^−/−^ mice had an empty stomach (Fig. 2C). They also had a very low glucose level (Fig. 2D), supporting strong hypoglycemia as the cause of death. To exclude a prenatal hypoglycemia, blood glucose levels were measured at E18.5 and were similar to wild type (Fig. 2E). Similar hypoglycemia was observed in the *Bin1*ex20skm^−/−^ mice (Fig. 2F,G). Altogether, these results show that BIN1 is necessary for muscle development and function, and for proper feeding after birth.

### BIN1 is necessary for triad formation and general muscle fiber organization

**Fig. 3.**
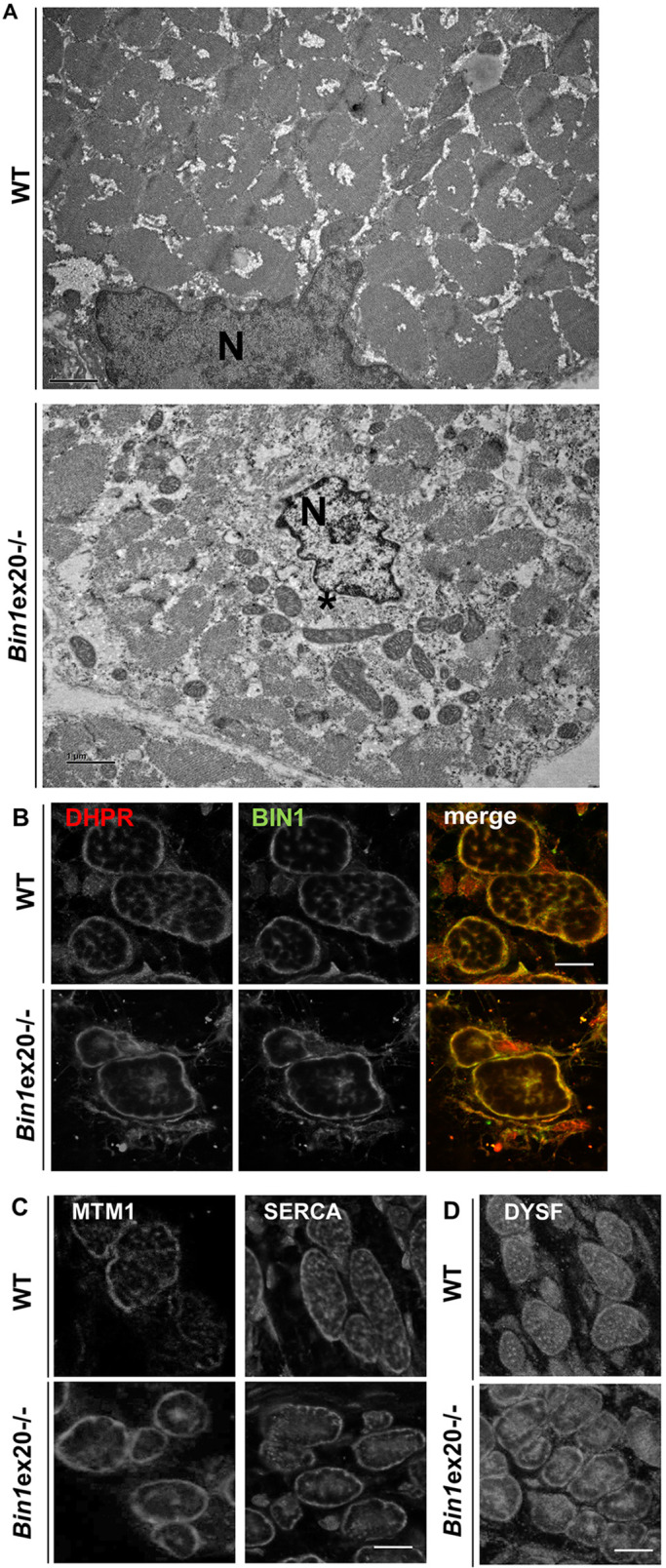
**Alterations in intracellular organization and triads upon BIN1 defect.** (A) Electron microscope visualized ultrastructure showed a general disorganization of muscle from newborn *Bin1*ex20^−/−^ mice with a central collapse of nuclei (N), surrounded by an area devoid of myofibrils and filled with mitochondria and amorphous materials (*). (B,C) BIN1 detected with a pan-isoform antibody colocalized with the T-tubule marker DHPR in newborn muscle fiber (transversal view). Markers of T-tubules (DHPR in B), junctional sarcoplasmic reticula (MTM1 in C) longitudinal sarcoplasmic reticula (SERCA in C) were collapsed to the center of myofibers in the *Bin1*ex20^−/−^ mice. (D) The marker of premature T-tubules (dysferlin) was collapsed to the center in *Bin1*ex20^−/−^ mice. Of note, muscle fibers in wild-type newborns are usually less polygonal than in adults. Immunolabeling of 8 µm transversal section. Scale bars: 1 µm (A); 10 µm (B-D).

To better understand the cellular defects due to BIN1 alteration, we performed histology, ultrastructure and immunolabeling analyses. Nicotinamide adenine dinucleotide dehydrogenase-tetrazolium reductase (NADH-TR) and succinate dehydrogenase (SDH) staining of *Bin1*ex20^−/−^ muscle sections showed a strong collapse of the oxidative activity towards the center of fibers (Fig. S3A,B). Alteration in mitochondria distribution was confirmed using an antibody against prohibitin, a mitochondria protein. Ultrastructure, visualized by transmission electron microscopy, confirmed the presence of amorphous material and collapsed mitochondria, and nuclei in the center of fibers that was deprived of myofibrils (Fig. 3A), reminiscent of a CNM-like histology (Toussaint et al., 2011).

In muscle transversal sections, BIN1 colocalized with the T-tubule channel protein dihydropyridine receptor (DHPR) in wild type, and residual truncated BIN1 aggregated in *Bin1*ex20^−/−^ along with DHPR (Fig. 3B). Newborn fibers appeared rounder than adult fibers. In addition, the SR markers SERCA1 and myotubularin1 (MTM1) were also collapsed toward the center of fibers in *Bin1*ex20^−/−^ muscles (Fig. 3C), supporting a general alteration of triads. Unlike triad markers, localization of DNM2 at the Z-line was not changed (Fig. S4).

**Fig. 4.**
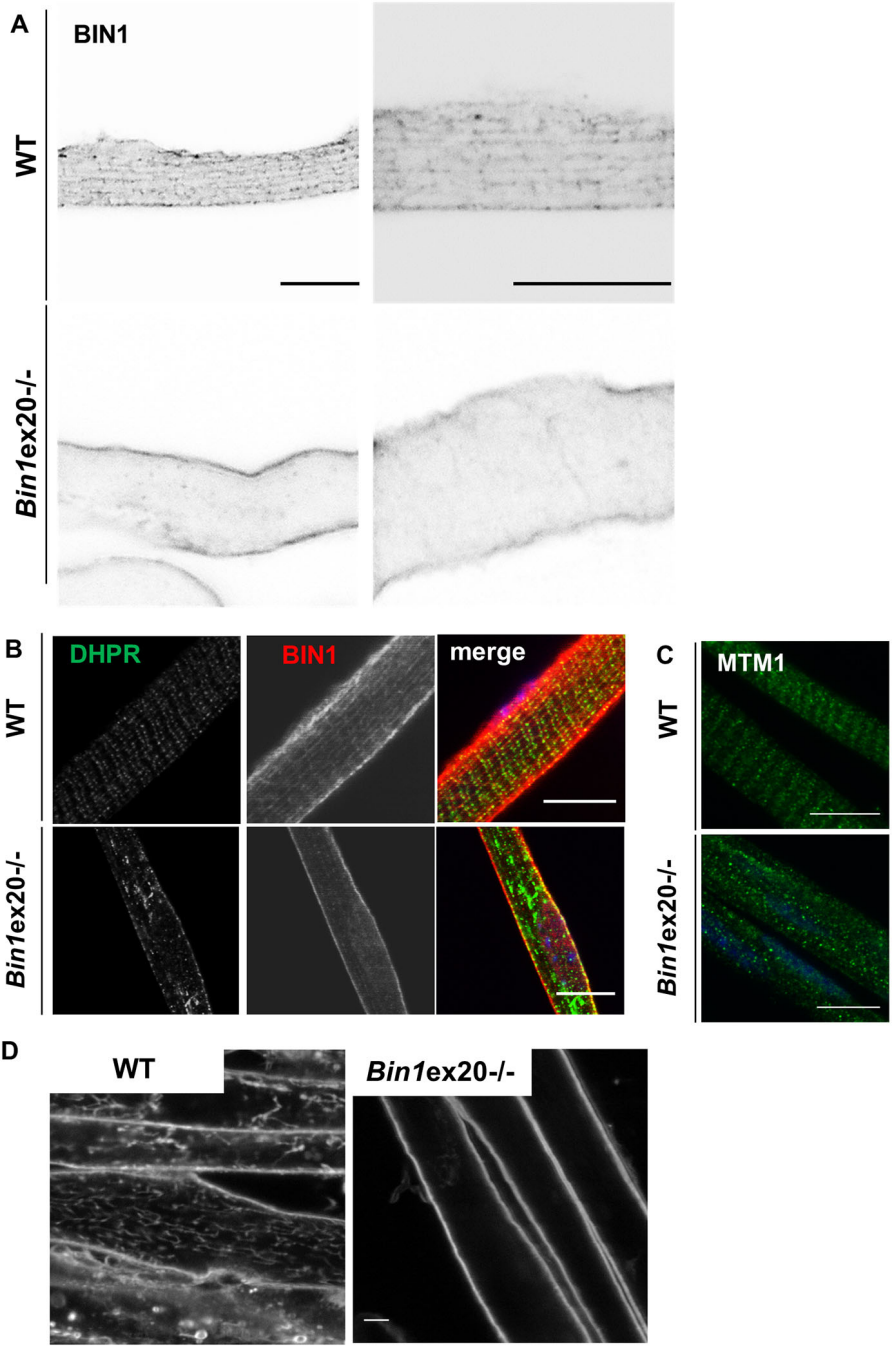
**Defects in T-tubule and triad formation.** T-tubule and BIN1 localization finish to mature around birth, and BIN1 is still partially longitudinal in newborn mice. (A) BIN1 detected with a pan-isoform antibody localizes on intracellular longitudinal tubules in newborn isolated myofibers, a pattern lost in *Bin1*ex20^−/−^ mice. Signal is shown in black over white. (B) DHPR aggregation in Bin1ex20^−/−^ isolated myofibers from newborn mice. (C) MTM1 localization was disrupted in *Bin1*ex20^−/−^ isolated myofibers. (D) Primary muscle cells differentiated into myotubes in culture and with FM4-64 staining of sarcolemma-connected membrane tubules; *Bin1*ex20^−/−^ myotubes lack the dense tubules network. Scale bars: 5 µm.

To investigate whether triad alterations were primarily due to a defect in T-tubule formation, we first localized in muscle sections from E18.5 embryos dysferlin, a protein implicated in T-tubule formation, and found it also collapsed toward the center of fibers, similarly to DHPR (Fig. 3D). Second, we validated that BIN1 localized as longitudinal and transversal tubules in isolated fibers from E18.5 embryos, a pattern lost in *Bin1*ex20^−/−^ (Fig. 4A,B). In isolated muscle fibers from E18.5 embryos, DHPR and MTM1 are normally localized at the T-tubules and I-band, respectively, whereas in the *Bin1*ex20^−/−^ both showed abnormal localization (Fig. 4B,C). Third, to assess the organization of T-tubules in formation, the FM4-64 impermeable lipophilic dye was applied to differentiated primary myotubes from wild-type and *Bin1*ex20^−/−^ mice. Although the T-tubules network was dense and developed in wild-type cultured myotubes, no labeled tubules were found in *Bin1*ex20^−/−^, suggesting nascent T-tubules were either missing or not connected to the plasma membrane and therefore not detectable with FM4-64 (Fig. 4D). These data support a primary defect in T-tubule formation, leading to alteration of the triad in mature muscle and impairment of organelle positioning.

### BIN1 is dispensable for adult muscle maintenance

To assess whether the effects of *Bin1* exon 20 deletion were dose dependent, *Bin1*ex20^+/−^ heterozygous mice were scrutinized. Heterozygous mice were found to be at the expected Mendelian ratio, had a normal lifespan and showed no obvious differences compared to their wild-type littermates. Body and muscle weight were comparable to wild type (Fig. S5A,B). *Bin1* heterozygous deletion did not affect the muscle performance assessed by the grip and rotarod tests (Fig. S5C,D). Muscle histology was normal as was T-tubule organization (Fig. S5E,F). Thus, the BIN1 defect does not have a dose-dependent effect in skeletal muscle, unlike in the heart in which it causes T-tubule defects and an increasing susceptibility to ventricular arrhythmias (Hong et al., 2014).

**Fig. 5.**
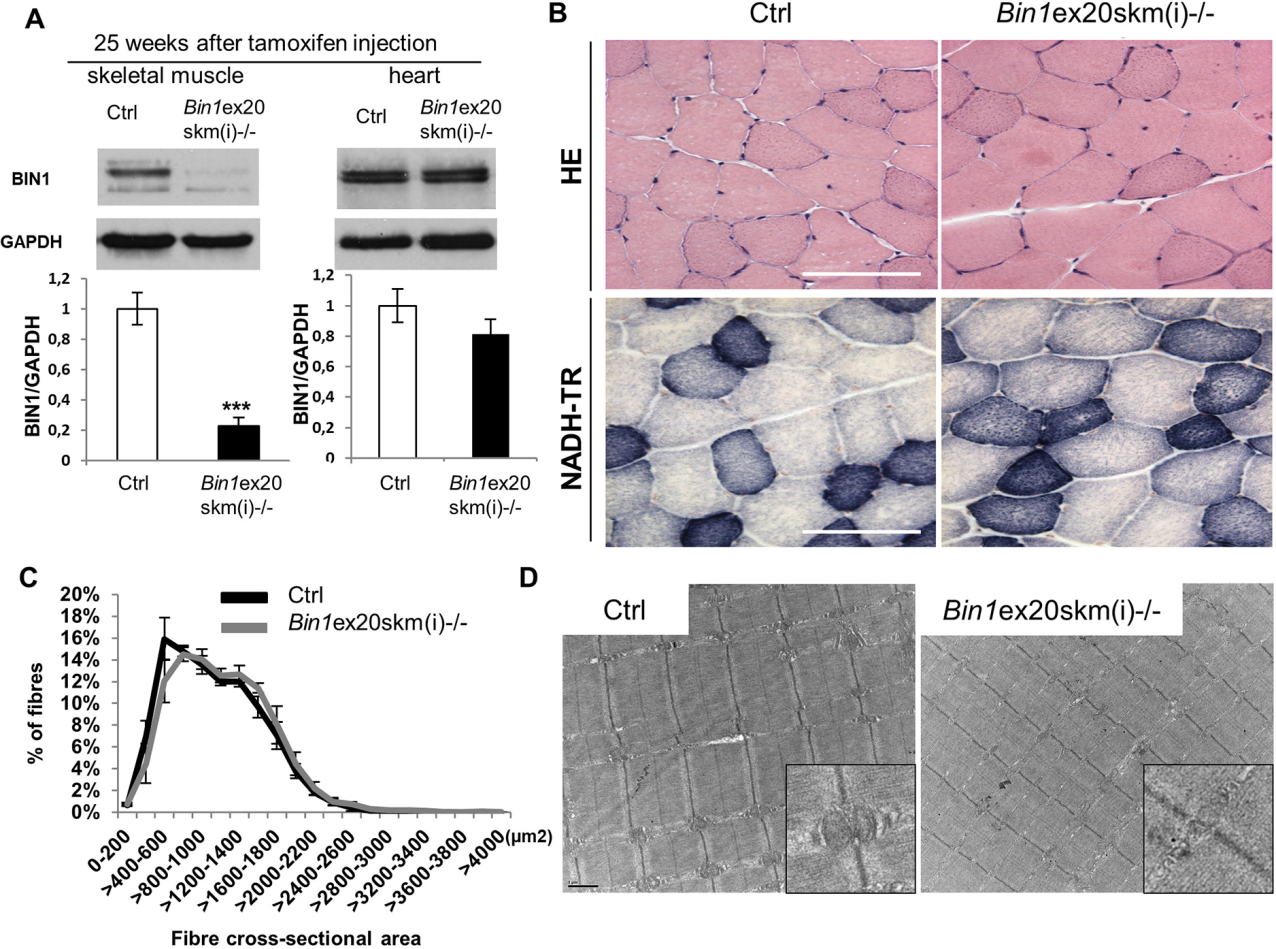
**BIN1 is not essential for adult muscle maintenance.** Characterization of *Bin1*ex20skm(i)^−/−^ mice 25 weeks after tamoxifen injection. (A) BIN1 protein level 25 weeks after intraperitoneal tamoxifen injection (*n*≥3 mice per group). Unpaired Student's *t*-test. ***P<0.001. (B) Histology of TA muscles stained with H&E (upper panel) and NADH-tetrazolium reductase (NADH-TR, lower panel). (C) Fiber cross-sectional areas of TA muscles were grouped into 200 µm² intervals, and represented as the percentage of total fibers in each group (*n*≥5 mice). (D) Electron microscopy images of TA muscles with zoomed images of triads (insets). Data are mean±s.e.m. Scale bars: 100 µm (B); 1 µm (D).

As deletion of *Bin1* exon 20 impacted on perinatal muscle development, we also investigated the importance of BIN1 in adult muscle maintenance. For this, *Bin1* exon 20 floxed mice were crossed with mice expressing a tamoxifen-inducible Cre recombinase under the control of the actin promoter to restrict *Bin1* deficiency specifically in adult muscle [*Bin1*ex20skm(i)^−/−^]. Following tamoxifen treatment at 7 weeks, *Bin1*ex20skm(i)^−/−^ mice were analyzed after 5 and 25 weeks. *Bin1* mRNA expression was strongly decreased after 5 weeks, with a 20% decrease in BIN1 protein level that reached 80% reduction at 25 weeks (Fig. 5; Fig. S6). Exon 20 deletion was skeletal muscle specific and did not impair BIN1 cardiac expression (Fig. 5A). We conducted a thorough investigation at 25 weeks and found no difference in body weight, muscle mass, grip, string and hanging tests in *Bin1*ex20skm(i)^−/−^ mice (Fig. S7). Histology with HE, NADH-TR and electron microscopy revealed no differences in fiber cross-sectional area or muscle organization (Fig. 5B-D). Taken together, these data showed that BIN1 is dispensable for adult muscle maintenance in unchallenged conditions.

### Removal of BIN1 muscle-specific isoforms does not affect muscle development and function

To understand the particular function of BIN1 muscle-specific isoforms encompassing exon 11 in muscle development and maintenance, we constitutively deleted this in-frame exon to obtain *Bin1*ex11^−/−^ mice expressing only ubiquitous BIN1 isoforms in skeletal muscle and mimicking *BIN1* exon 11 skipping found in myotonic dystrophy, and in the highly progressive form of CNM (Fugier et al., 2011; Böhm et al., 2013). Specific excision of exon 11 in muscle, without alteration in the splicing of neighboring exons, was confirmed by RT-PCR and sequencing of the corresponding cDNAs (Fig. S8A-D). Note that adult muscle contains two BIN1 isoforms that differ by the presence of alternative exon 17 (iso8±exon 17; Table S1) (Cowling et al., 2017). Western blot with muscle extracts using an antibody against the PI domain (encoded by exon 11) detected no signal (Fig. S8E). Other BIN1 isoforms were still detected with a pan-antibody raised against the SH3 domain. Removal of the *Bin1* muscle-specific isoform caused no detectable upregulation of amphiphysin 1 (Fig. S8F).

**Fig. 6.**
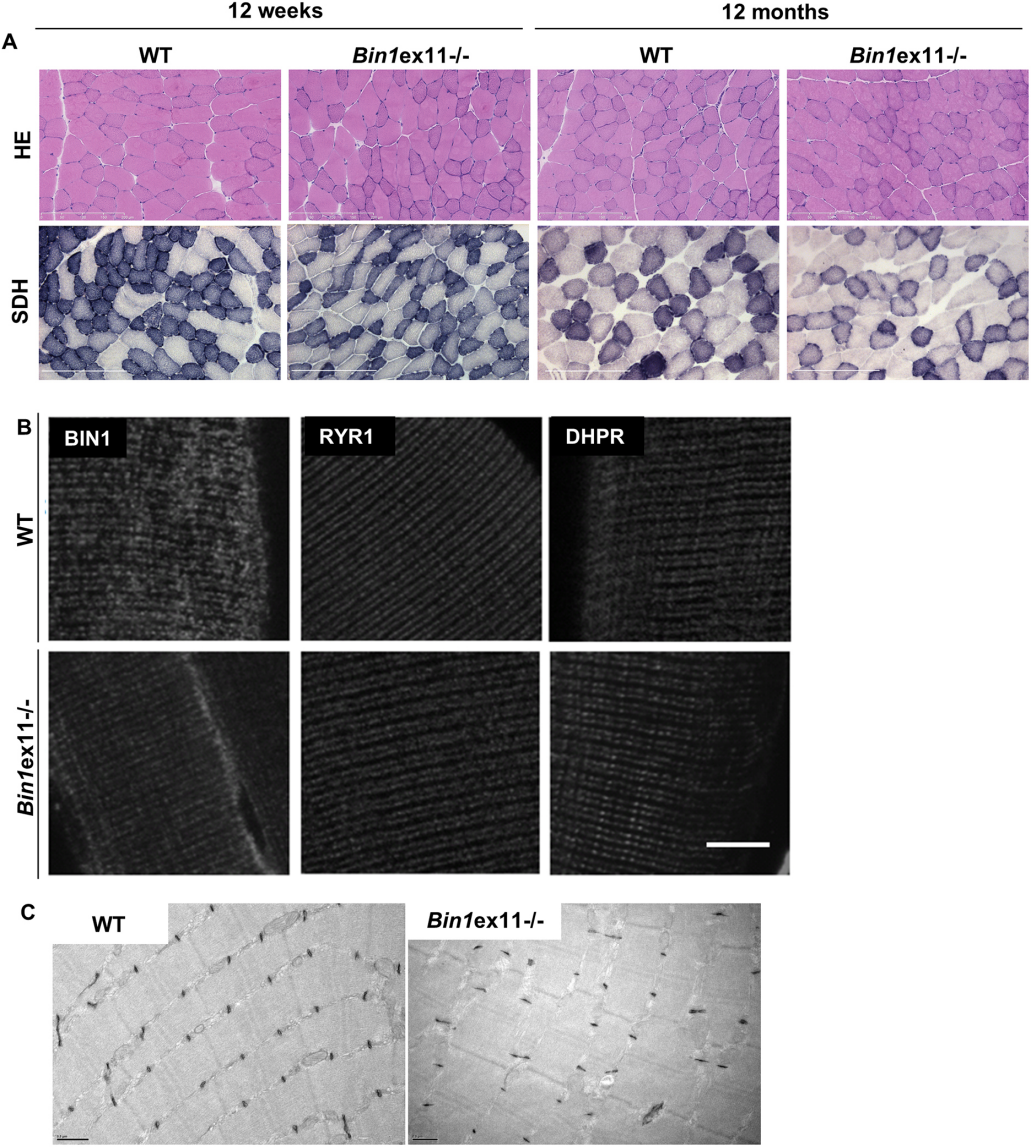
**Lack of BIN1 muscle-specific isoforms correlates with normal histology and triad structure.** (A) Histological features in *Bin1*ex11^−/−^ mice at 12 weeks and 12 months of age with H&E or SDH. (B) Localization of BIN1 without the PI domain and triad markers in isolated fibers stained with BIN1 (pan-isoform antibody), RYR1 (sarcoplasmic reticula), or DHPR (T-tubules) antibodies. (C) Electron micrograph of 12-week-old wild-type (WT) and *Bin1*ex11^−/−^ muscles labeled with potassium ferrocyanide for T-tubules. Scale bars: 200 µm (A); 10 µm (B); 0.5 µm (C).

*Bin1*ex11^−/−^ mice were born following the expected Mendelian ratio and had normal lifespans, supporting the notion that the PI domain is dispensable for muscle development. Phenotyping young adults at 12 weeks showed no difference in wild type in body weight and length, muscle weight or fat and lean mass distribution (Fig. S9A-D). Deletion of exon 11 neither impacted motor performance, as measured using grip and rotarod tests, nor reduced specific muscle force (Fig. S10A-D). Muscle histology (H&E and SDH stainings), fiber cross-sectional area and muscle ultrastructure were comparable between *Bin1*ex11^−/−^ and wild-type mice (Fig. 6A-C; Fig. S11). Only nuclei mislocalization was slightly but significantly increased from 1% in wild type to 3.7% in *Bin1*ex11^−/−^ animals in the TA (Fig. S11B). We also checked whether the loss of muscle-specific BIN1 isoforms had any impact on T-tubule and triad organization. BIN1 without the PI domain still localized to the triad. Immunofluorescence and potassium ferrocyanide revealed normal T-tubule structure and organization in *Bin1*ex11^−/−^ muscle (Fig. 6B,C).

**Fig. 7.**
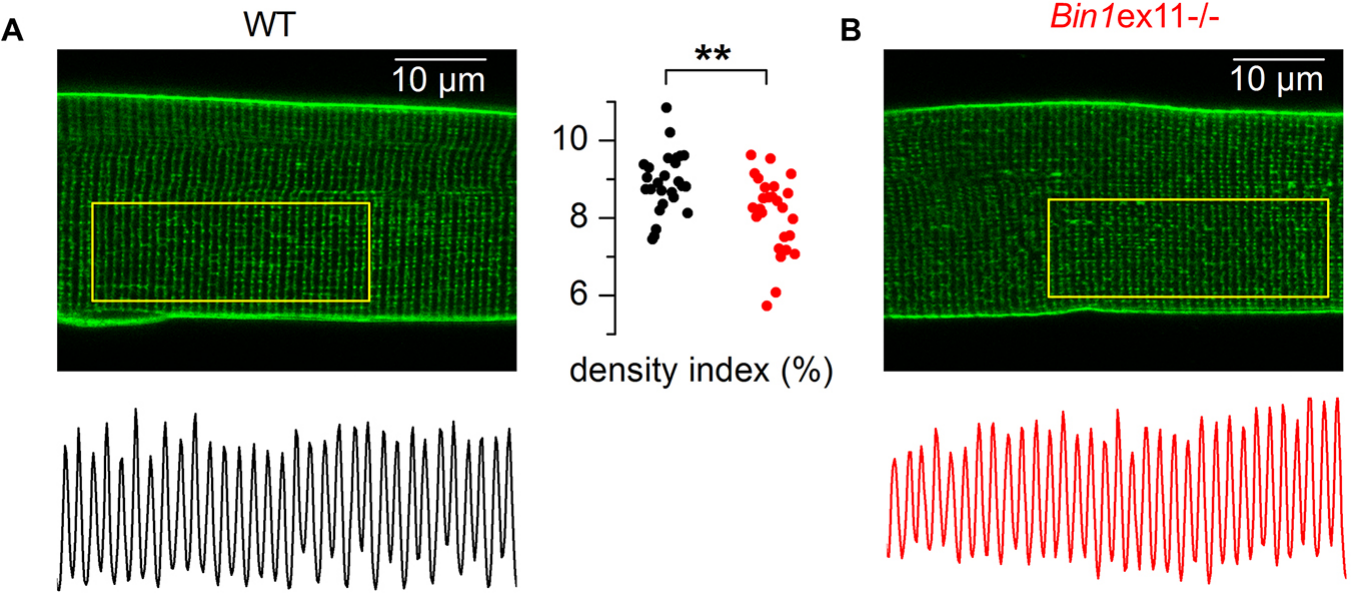
**T-tubule network in muscle fibers from WT and *Bin1*ex11^−/−^** **mice.** (A,B) *x*,*y* fluorescence images of di-8-anepps staining in fibers from a wild-type (WT) mouse (A) and from a *Bin1*ex11^−/−^ mouse (B). The longitudinal profile of fluorescence along the outlined region of interest is shown below each image. The graph in the middle shows values for the T-tubule density index in fibers from wild-type mice (black points) and *Bin1*ex11^−/−^ mice (red points). Unpaired Student's *t*-test. **P<0.01.

Di-8-anepps staining revealed a slight reduction of the T-tubule network density (Fig. 7A,B), although average mean values for membrane capacitance of the portion of fiber under study did not differ between fibers from *Bin1*ex11^−/−^ and wild-type mice (1.45±0.09 nF, *n*=19 fibers, versus 1.59±0.13 nF, *n*=18 fibers, respectively). To assess in more detail whether there was a functional defect of the EC coupling in *Bin1*ex11^−/−^ mice, we analyzed the functional properties of DHPR and of the ryanodine receptor, RYR1, in intact isolated muscle fibers. Analysis of the voltage dependence of DHPR Ca^2+^ current density revealed no change in maximum conductance, apparent reversal voltage, steepness factor and half-activation voltage in the *Bin1*ex11^−/−^ group (Fig. S12). Regarding RYR1 activity, analysis of the voltage dependence of the peak rate of SR calcium release revealed no significant difference in maximum amplitude, time to peak, voltage for half activation and steepness factor between wild type and *Bin1*ex11^−/−^ fibers (Fig. S13). Voltage-activated Ca^2+^ transients were also measured with the low affinity dye fluo-4 FF, in the absence of exogenous intracellular Ca^2+^ buffer, to assess the myoplasmic Ca^2+^ removal capabilities of the fibers. Fitting a single exponential function to the decay of the fluo-4 FF transients showed no difference between mean values in the two groups, suggesting no change in Ca^2+^ buffering and SR calcium uptake rate by SERCA pumps (Fig. S14). Overall, the PI domain appears dispensable for myofiber structure and function.

Long-term deletion (12 months of age) of exon 11 caused no additional phenotypes than at 12 weeks. In particular, the histology was similar at both ages with normal fiber size and a slight increase in nuclei mislocalization compared to wild type (Fig. 6A; Fig. S11B), and the specific TA muscle force was normal (Fig. S15). However, we noted at 12 months a slight and significant increase in body weight and fat mass in *Bin1*ex11^−/−^ mice (Fig. S15). At that age, the intracellular organization of muscle fibers was normal (Fig. S16). Mouse muscles mature until 7 weeks of age (Engel and Franzini-Armstrong, 2004). To see whether there were any early defects during postnatal muscle maturation in *Bin1*ex11^−/−^ mice, we examined the mice 2 weeks after birth. Body weight and muscle mass were comparable to wild type (Fig. S17A,B). Histology revealed no anomaly, and there was no difference in fiber size, nuclei localization, sarcomere and T-tubule organization (Fig. S17C-F). In conclusion, although BIN1 ubiquitous isoforms are mainly implicated in muscle development, the muscle-specific isoforms appear dispensable for muscle formation and maintenance, raising the question of their specific function.

### The BIN1 muscle-specific isoforms are beneficial for muscle regeneration

**Fig. 8.**
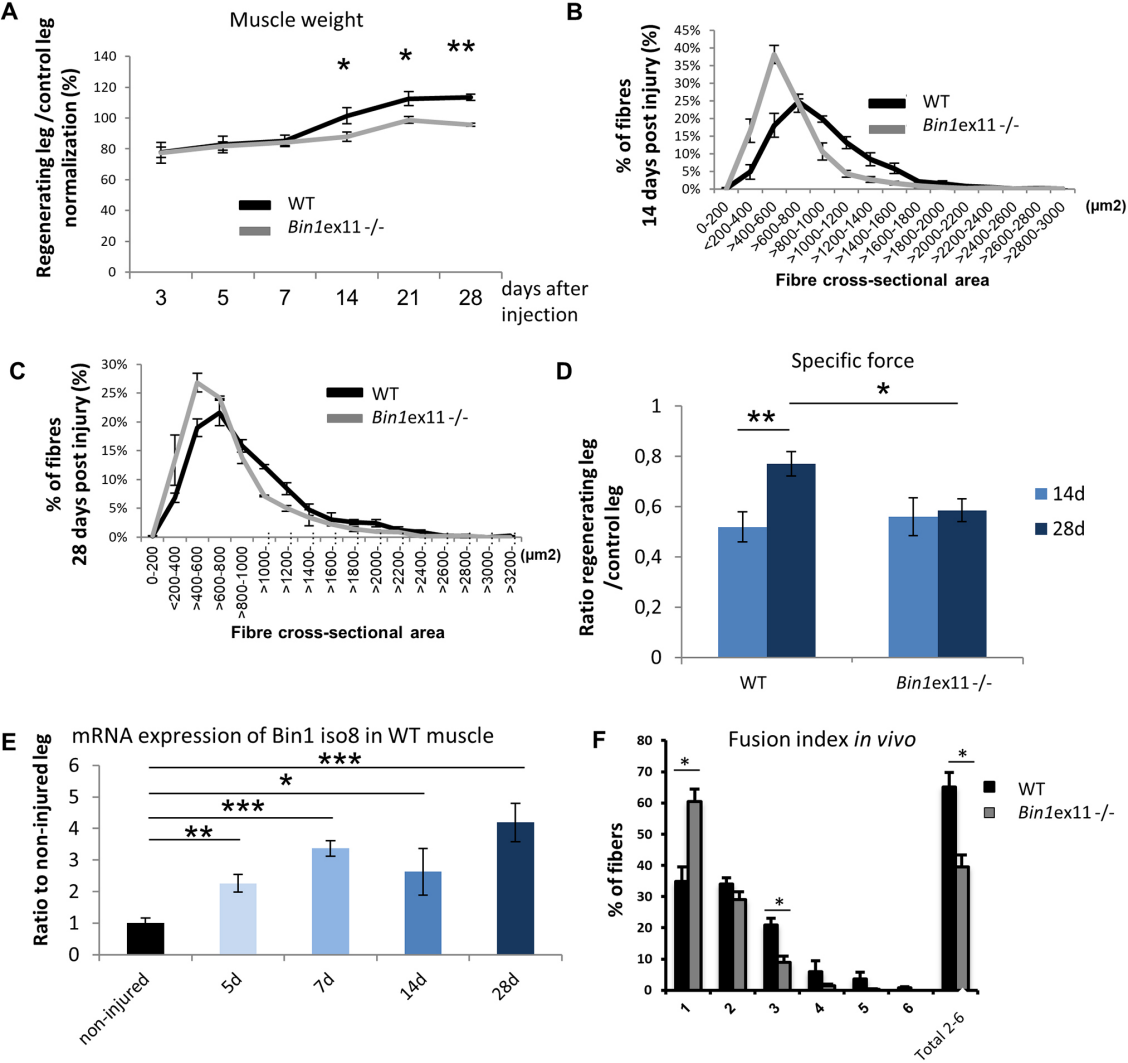
**Muscle regeneration is altered in *Bin1*ex11^−/−^** **mice.** Right TA muscles of 12 weeks mice were injected with notexin and left TA muscles were used as control. (A) Regenerative capacity, estimated through normalization of the muscle mass of injected leg to uninjected contralateral leg at different time points; represented as the percentage of recovery. (B,C) TA muscle fiber cross-sectional area at 14 days (B) and 28 days (C) after notexin injection. Only regenerating fibers (with centralized nuclei) were taken into account and fiber cross-sectional area is grouped into 200 µm² intervals, and represented as the percentage of total fibers (*n*=5-7 mice). (D) Specific maximal force of TA muscles expressed as the ratio between regenerating and control legs. Unlike wild-type (WT), *Bin1*ex11^−/−^ muscle force does not increase with time. (E) *Bin1* iso8 (muscle-specific) mRNA expression in wild-type mice in non-injured muscle and during regeneration. (F) Fusion index 14 days after notexin injection in wild-type and *Bin1*ex11^−/−^ TA muscles. Number of nuclei per fiber was counted on transversal sections. *n*=3 mice per group. Data are mean±s.e.m. Unpaired Student's *t*-test. *P<0.05,**P<0.01,***P<0.001.

The only phenotype observed in *Bin1*ex11^−/−^ mice in young and old mice was a slight increase in nuclei centralization, a feature observed during normal muscle regeneration. To test the ability of *Bin1*ex11^−/−^ muscle to regenerate, TA muscles were injected with notexin to produce muscle damage and analyzed 3, 5, 7, 14, 21 and 28 days after the injury. Normalization of the recovery of the damaged muscle towards the contralateral control muscle showed that, at the later stages of regeneration (14 and 28 days), the weight of the damaged *Bin1*ex11^−/−^ muscle significantly failed to recover compared to wild type (Fig. 8A). Histological examinations and quantification confirmed it was correlated to smaller fiber cross-sectional area (Fig. 8B,C; Fig. S18). The specific muscle force showed a lag in the recovery at 28 days, supporting the notion that muscles without BIN1 muscle-specific isoforms produced intrinsically less force than wild-type muscles at the late stage of muscle regeneration (Fig. 8D).

To validate the implication of BIN1 muscle-specific isoforms in muscle regeneration, the expression of BIN1 isoforms encompassing exon 11 was investigated by RT-qPCR and found to be increased at different time points during regeneration (Fig. 8E). To investigate potential cell fusion defects that may explain the deficit in early muscle growth during regeneration in the *Bin1*ex11^−/−^ mice, nuclei counting was performed in regenerating myofibers and revealed a decreased mean number of nuclei per fiber in *Bin1*ex11^−/−^ muscle (Fig. 8F), correlating with the observed reduction in fiber cross-sectional area and the specific muscle force. This *in vivo* alteration was not due to an intrinsic defect in fusion or differentiation abilities as primary myoblasts from *Bin1*ex11^−/−^ mice had a normal fusion and differentiation index when differentiated *in vitro* (Fig. S19). Of note, the number of PAX7^+^ cells was decreased in *Bin1*ex11^−/−^ muscle compared to wild type (Fig. S20), suggesting that the defective fusion events found *in vivo* result from a reduced pool of satellite cells. In conclusion, BIN1 muscle isoforms appear to be important for muscle regeneration but not for muscle development.

## DISCUSSION

In *Bin1*ex20^−/−^ mice, the SH3 domain present in all isoforms was disrupted, similar to some CNM patients with truncating mutations. This led to a strong decrease in BIN1 level and to perinatal death, primarily involving muscle defects. Conversely, *Bin1*ex11^−/−^ mice, in which the muscle-specific isoforms were converted into ubiquitous isoforms by deletion of the in-frame exon 11 coding for the PI domain, had a normal development and muscle function. However, following muscle damage promoted by notexin injection, these mice displayed a delayed muscle regeneration with a defect in myoblast fusion. This study shows that BIN1 is a crucial player in muscle development and that perinatal muscle maturation and function are affected in its absence. In addition, absence of its skeletal muscle-specific isoforms in mice does not alter muscle development but leads to impaired muscle regeneration in adult.

### BIN1 ubiquitous isoforms and skeletal muscle development

Our data confirmed that *Bin1* has the highest expression level in skeletal muscle (Butler et al., 1997; Wechsler-Reya et al., 1997). *Bin1*ex20^−/−^ mice die within the first few hours after birth. A previously published total *Bin1* KO mice also died at birth, possibly from a suspected cardiomyopathy, with no reported defects of skeletal muscle structure (Muller et al., 2003). However, mice with cardiac-specific BIN1 loss survive to adulthood (Hong et al., 2014; Laury-Kleintop et al., 2015). In our *Bin1*ex20^−/−^ model, thorough analysis of heart structure and function revealed no differences compared with wild type. Together with the observation that skeletal muscle-specific deletion of exon 20 displayed the same lethality as the constitutive deletion, we conclude that postnatal lethality is due to skeletal muscle impairment.

The first *in vivo* study investigating the role of amphiphysins in muscle development was carried out with *Drosophila*. An amphiphysin null mutant was viable but flightless, and had major T-tubule malformations (Razzaq et al., 2001). There are two amphiphysins in mammals (AMPH1 and BIN1). We show here that mammalian BIN1 has kept a similar function as its *Drosophila* ortholog. In *Bin1*ex20^−/−^ mice, the muscle developmental defects are correlated with triad disorganization, as well as a major DHPR (T-tubule marker) collapse. These structural defects originate from a lack of T-tubule formation as there were no T-tubules connected to the plasma membrane in *Bin1*ex20^−/−^ primary myotubes and the localization of dysferlin, a main regulator of T-tubule formation, was impaired in newborn *Bin1*ex20^−/−^ muscle. Moreover, we specifically localized BIN1 on triads, in agreement with previous data (Butler et al., 1997). The intracellular tubule network observed by BIN1 immunofluorescence in primary myotubes and isolated myofibers is connected to the membrane, therefore strongly supporting a role for BIN1 in T-tubule formation, as suggested in C2C12 cultured myotubes (Lee et al., 2002). A junctional reticulum defect may be a consequence of the absence of T-tubules, as anchoring of the T-tubule with the SR is essential for the maturation of the whole triad and subsequently the muscle fiber (Flucher et al., 1993). In conclusion, BIN1 is not necessary for early myogenesis but is essential for muscle development and perinatal survival through its role in skeletal muscle T-tubule formation. Importantly, we showed that BIN1 is also essential for the general organization of myofibers. BIN1 deficiency also led to mitochondria and nuclei mispositioning in muscle. BIN1 appears critical for nuclear positioning, as we previously showed BIN1 binds to nesprin, a nuclear envelope protein, and that lack of the BIN1 ortholog *amph-1* in *Caenorhabditis elegans* resulted in phenocopying of the nuclear mispositioning of nesprin mutants in non-muscle cells (D'Alessandro et al., 2015). However, a direct impact of BIN1 on mitochondria position remains to be deciphered.

### BIN1 muscle-specific isoforms and muscle regeneration

To address whether BIN1 also has a role in skeletal muscle maintenance, muscle-specific ablation of *Bin1* was performed in adults at 7 weeks of age using tamoxifen activation of the Cre recombinase under the control of the skeletal actin promoter. Our data showed that even 25 weeks after the induction of the deletion and although BIN1 protein level showed a strong reduction, the analyzed muscle displayed no difference in histology, ultrastructure or motor function, including calcium homeostasis. These data differ from shRNA knockdown of *Bin1* in adult muscle fibers in which ∼30% of muscle fibers showed swollen T-tubules and greatly reduced Ca^2+^ sparks frequency 14 days after the shRNA electroporation (Tjondrokoesoemo et al., 2011). It is possible that electroporation induced muscle regeneration, which we show to be dependent on BIN1.

The muscle-specific exon 11 is upregulated during muscle differentiation both *in vitro* and *in vivo*, and is present in most muscle isoforms in mature murine and human muscle (Butler et al., 1997; Wechsler-Reya et al., 1997; Nicot et al., 2007; Fugier et al., 2011; Cowling et al., 2017). Our *Bin1*ex11^−/−^ model did not have significant defects in T-tubule structure at each of the time points tested (2 weeks, 12 weeks and 12 months of age). Our findings are in apparent contradiction with the *in vitro* data showing that the PI domain is necessary for membrane tubulation in cultured cells (Lee et al., 2002; Nicot et al., 2007; Böhm et al., 2013). Our data indicate that the PI domain is neither required for T-tubule formation nor muscle development. Similarly, alterations of muscle development in bin1 zebrafish morphants were rescued with human BIN1 independently of the presence of the PI domain (Smith et al., 2014). Potentially, unidentified regulators of membrane tubulation present *in vivo*, but absent in cultured cells, may explain this discrepancy. Among the candidates are BIN1 interactors or modifiers of membrane physicochemical properties. Indeed T-tubule defects have been reported in a canine CNM (Walmsley et al., 2017), a model characterized by an altered lipid composition and fluidity of muscle membrane (Blondelle et al., 2015).

Here, we reveal a role for the muscle-specific isoforms of BIN1 in muscle regeneration. We used notexin-induced muscle regeneration in *Bin1*ex11^−/−^ mice and observed a decrease in fiber cross-sectional area at 14 and 28 days after the muscle damage associated with a reduced fusion index and eventually led to a decrease in specific force recovery. A deficit in myofiber growth during early regeneration was previously linked to a defect in myoblast fusion (Millay et al., 2014; Baghdadi and Tajbakhsh, 2018). Additionally, BIN1 was abundant at the fusion sites of myotubes (Klinge et al., 2007), and C2C12 cells expressing antisense *Bin1* showed an impaired myoblast fusion (Wechsler-Reya et al., 1998). We cannot exclude a membrane repair component as shRNA-mediated downregulation of *Bin1* compromised myofiber membrane integrity (Tjondrokoesoemo et al., 2011) and BIN1 was located close to the repair cap (Demonbreun et al., 2016). However, BIN1 was never directly implicated in muscle membrane repair and creatine kinase levels were comparable to wild type after treadmill exercise in *Bin1*ex11^−/−^ mice (757±252 U/l versus 773±288 U/l). Overall, BIN1 is necessary for muscle development and function, whereas the PI domain requirement is restricted to muscle regeneration.

### Physiopathology of centronuclear myopathies

In humans, *BIN1* mutations lead to autosomal recessive CNM with neonatal onset (Nicot et al., 2007) or autosomal dominant CNM with adult onset (Böhm et al., 2014). Exon 11 skipping causes childhood onset progressive CNM (Böhm et al., 2013), and the mis-splicing of exons 7 and 11 was identified in myotonic dystrophy (Fugier et al., 2011).

*Bin1*ex20^−/−^ display a strong decrease of BIN1 when its SH3 domain is deleted. Recessive CNM patients have either a truncated SH3 domain or missense changes in the BAR domain (Nicot et al., 2007; Böhm et al., 2010; Claeys et al., 2010). They exhibit hypotonia at birth and delayed motor development, and several patients died soon after birth. General histology showed highly variable fiber cross-sectional area with a high number of centralized nuclei often clustered, and NADH-TR staining showed oxidative staining aggregations (Romero, 2010; Toussaint et al., 2011). The majority of triads had swollen T-tubules or SR, and DHPR accumulated in the center of the fiber, disclosing a strong collapse of the T-tubule network. The *Bin1*ex20^−/−^ mouse reproduces these histological hallmarks and the muscle impairment, and therefore represents a faithful model to study the mechanisms of recessive CNM. Using this model, we found alterations of the T-tubule formation that could be at the basis of the CNM pathology. BIN1 could potentially regulate DNM2, another protein mutated in CNM, as DNM2 was proposed to modulate T-tubule formation or maintenance in muscle development in *Drosophila* (Chin et al., 2015). Indeed, CNM mutations truncating the BIN1 SH3 domain strongly impaired binding to DNM2 (Nicot et al., 2007), and DNM2 downregulation rescued the perinatal death of *Bin1*ex20^−/−^ mice (Cowling et al., 2017). Of note, the *Bin1*ex20± heterozygous mice did not display phenotypes, unlike CNM patients with dominant mutations, suggesting dominant CNM is not a result of BIN1 haploinsufficiency but of a gain-of-function mechanism.

Under unstressed conditions, the *Bin1*ex11^−/−^ mice did not develop a CNM phenotype, except for a slight but significant increase in nuclei mislocalization. Recessive CNM patients were previously identified with a splice site mutation in exon 11, in which exon 11 was removed without BIN1 levels being changed (Böhm et al., 2013). Affected patients had normal motor development. Muscle weakness was noticed at 3.5 years of age, after which the disease became highly progressive and two out of three patients died at the age of 5 and 7 years, whereas the third was wheelchair bound. They had a classical CNM histology. *Bin1*ex11^−/−^ mice displayed a muscle regeneration defect that could be an important trigger for the patient phenotypes as they were not affected at birth but rather affected by a progressive muscle weakness.

Exon 11 is also mis-spliced in DM, together with abnormal inclusion of brain-specific exon 7 (Fugier et al., 2011). DM is a pleiotropic disease including gradually worsening muscle loss and weakness, and a CNM-like histology. Unlike in the *Bin1*ex11^−/−^ mice, specific exon 11 skipping using a U7 snRNA method in adult mice showed a significant reduction of muscle force together with extensive T-tubule disorganization (Fugier et al., 2011). One cannot exclude some U7 off-target effects, or conversely a compensatory mechanism during fetal development in the *Bin1*ex11^−/−^ mice. Our data suggest that DM muscle phenotypes might be mainly due to exon 7 inclusion rather than exon 11 skipping. Of note, exon 7 inclusion in BIN1 promoted binding to DNM2 (Ellis et al., 2012), again suggesting that the interplay between BIN1 and DNM2 is important for normal muscle function and in the setup of the CNM and DM myopathies.

In conclusion, BIN1 ubiquitous function is necessary for muscle development and function, whereas BIN1 muscle-specific isoforms are implicated in muscle regeneration. This supports the notion that BIN1 CNM with congenital onset is due to developmental defects, whereas CNM forms with later onset may be due to regeneration defects.

## MATERIALS AND METHODS

### Materials

Primary antibodies used were: mouse anti-DHPRα1 (Cav 1.1) subunit (Affinity Bioreagents, MA3-920 or Abcam, ab58552), α-actinin (Sigma-Aldrich, EA-53), BIN1 C99D clone (Sigma-Aldrich, B9428), RYR1 (clone 34C; Sigma-Aldrich, R129) DNA polymerase (Santa Cruz Biotechnology, sc-373884) and glyceraldehyde-3-phosphate dehydrogenase (GAPDH; Chemicon, MAB374) monoclonal antibodies; and rabbit anti-RYR1 (a kind gift from Isabelle Marty, Grenoble Institut des Neurosciences, France). Rabbit anti-DNM2 antibodies (R2680 and R2865) (Cowling et al., 2014), anti-MTM1 (R2827) (Hnia et al., 2011) and anti-BIN1 (R2444 against the SH3 domain and R2405 against the PI domain) (Nicot et al., 2007) were made onsite at the antibody facility of IGBMC. Alexa-conjugated secondary antibodies were purchased from Invitrogen. Secondary antibodies against mouse and rabbit IgG, conjugated with horseradish peroxidase (HRP) were purchased from Jackson ImmunoResearch. The following products were purchased: Hoechst nuclear stain (Sigma-Aldrich, B2883), an ECL chemiluminescent reaction kit (Pierce), LipofectamineTM (Life Technologies), Tamoxifen (Sigma-Aldrich) and Notexin (Latoxan).

### Generation of *Bin1* exon 11- and *Bin1* exon 20-deleted mice

The targeting vector was created with LoxP sites flanking exon 11 of *Bin1* or exon 20, then linearized and electroporated into embryonic stem cells (ESCs). Recombinant ESCs were injected into C57BL/6 blastocysts that were implanted in pseudo-pregnant females and the germline transmission was validated. Recombination was triggered using transgenic mice expressing the Cre recombinase under the control of the cytomegalovirus or ACTA1 (human skeletal actin, HSA) promoters. B6J strain mice were bred and analyzed. In case of time-inducible recombination, the *HSACreERt2* transgenic line was used (Miniou et al., 1999). To trigger the recombination, 7-week-old mice were injected daily for 5 consecutive days with 100 µl of tamoxifen solution diluted in 90% vegetable oil and 10% ethanol at 1 mg/100 µl.

### Animal general phenotyping

Animal experimentation was approved by the institutional ethical committee Com'Eth IGBMC-ICS (2012-128; 4469). Animals were housed in a temperature-controlled room (19-22°C) with a 12:12-h light/dark cycle. Mice were weighed weekly until 1 year of age. Mice were humanely killed when required by CO_2_ inhalation followed by cervical dislocation, according to national and European legislation on animal experimentation. Mice aged 10-15 weeks were phenotyped under the European Mouse Disease Clinic (EUMODIC) phenotyping program (www.eumodic.eu), with results made publicly available (www.europhenome.org). Male and female mutant mice were compared to wild-type littermates. Blood chemistry, echocardiography, Dexascan presented here for male mice (*n*= minimum 10 per group) were performed as part of pipelines 1 and 2 of the EUMODIC phenotyping program at the Institut Clinique de la Souris (www.ics-mci.fr). Blood glucose concentration was determined with a glucometer and test strips (One touch ultra), both purchased from Lifescan.

### Motor function

The following procedure was carried out for the string test: mice were suspended on a wire by their forelimbs, and allowed 20 s to climb their hindlimbs onto the wire. Three trials per mouse were performed, with 5 min rest between trials. A fall was considered equal to 20 s (*n*≥5 mice per group). Grip strength testing was performed by placing the two front paws or all four paws on the grid of a dynamometer (Bioseb) and pulling mice by the tail in the opposite direction. The maximal strength exerted by the mouse before losing grip was recorded. Three trials per mouse were performed, with 30 s rest between trials (two paws test, *n*≥5 mice per group; four paws test, *n*=5-7 mice per group). For the hanging test, mice were suspended from a cage lid for a maximum of 60 s. The amount of time before the mouse fell off the cage was recorded for each trial. Three trials per mouse were performed. For the rotarod test, coordination and whole-body muscle strength and fatigability were tested using an accelerated rotating rod test (Panlab). Mice were placed on the rod which accelerated from 4 to 40 rpm in 5 min. Three trials per day, with 5 min rest between trials, were performed for day 1 (training day) and then for a further 4 days during which results were recorded. Animals were scored for their latency to fall (in seconds). The mean of the three trials was calculated for each experiment listed above (*n*=5-7 mice per group).

### Echocardiography

Pregnant mice were studied on days 17.5 to 18.5 of gestation (E17.5 to E18.5) (where 18.5 days is full term), and a total of 19 wild-type and KO embryos were observed. A Vevo 2100 (VisualSonics) system with a 40 MHz transducer (lateral and axial resolutions of 68 and 38 μm, respectively) was used for ultrasound interrogation of the embryos. Pregnant female mice were anesthetized with isoflurane (1.5% isoflurane in medical air containing 21% oxygen) and laid supine in a Petri dish filled with physiological solution, with the right forearm and left hindlimb implanted with electrocardiogram electrodes subcutaneously for heart rate monitoring (450-550 beats/min). Body temperature and physiological solution temperature were both monitored via a thermometer (rectal thermometer for body temperature) and maintained at 36 to 38°C using a lamp (for the mouse) and a heating pad (for the saline solution). The obtainment of a proper imaging plane involved externalization of the uterus into the warm physiological solution bath followed by placement of the transducer directly on the uterine wall where prewarmed ultrasound gel was applied. M-mode imaging produced images that provided the most accurate measurements of ventricular wall thickness and shortening fraction in fetal mouse heart, measuring 2-5 mm. In this mode, the improved frame rate of up to 1000 frames/sec allowed end systole and end diastole to be measured with ease. At the end of the procedure, the pregnant mouse was killed by cervical dislocation and biopsies of the fetuses were taken for genotyping.

### Muscle contractile properties

*In situ* muscle isometric contraction was measured in response to nerve and muscle stimulation, as described previously with a force transducer (Vignaud et al., 2005, 2010). Results from nerve stimulation are shown (*n*=5-11 mice per group). After contractile measurements, the animals were killed by cervical dislocation. TA muscles were then dissected and weighed to determine specific maximal force.

### *In vivo* muscle regeneration test

Twelve-week-old male *Bin1*ex11^−/−^ and wild-type mice were used. A cycle of degeneration and regeneration was induced and studied in the TA by intramuscular injection of 20 µl notexin diluted in PBS to 10 g/ml in the right leg. The contralateral TA muscle was used as a control. At various time points after the injections, mice were killed by cervical dislocation. The TA muscles of both hindlimbs were immediately removed, snap frozen in isopentane cooled in liquid nitrogen, mounted in OCT, and 8 µm transversal sections were cut and mounted onto SuperFrost Plus slides.

### *In situ* hybridization

*In situ* hybridizations were performed on whole-mount wild-type embryos at E14.5 and E18.5 (Diez-Roux et al., 2011). The probe used for BIN1 was designed using the Eurexpress program (www.eurexpress.org/ee). The BIN1 probe sequence is as follows: TTTTTTTTTTTTTTTTTGGCTTTGGCAGGTTTTTCTTTTTTGTTTGTTTCGCTGCATTTTGAACACTAGGGCTTATTTTCAAACAGCACACGGTTGGTCTGCAGAGCGGAACCAGGCTGGGCCAGTGTGCAGGCCCTGCCCAGGGCAGCTGCCAGAAGAGGACCCAAGCCCTGCTCGGTGGCGCAGCCAAGCGTCAGGCAAGTGTGGCGGTGGCTCTGCACCCCCGGCCCGCCCTGAACATGCGGGCATCGGGAACTCAACTAGGGGGGGACACAGCAGCTTCAGGAACACTGGAGAAGTCCACTGAACGGGGTCGGACGGCTCTTTGGAAAACCACCTCATCTTTGGGGGTATACCTCTTGGGTTAGGTTTCCGCCCCCCAGTTTCCCGACACTTTTCAAGAATTTCACAACAAAACAGAAACAGAAAAGAGAG.

### Primary cell culture

Primary myoblasts were collected from mice as described before (Falcone et al., 2014). Briefly, hindlimb muscles from 6-day-old pups were extracted and digested with collagenase (Sigma-Aldrich, C9263-1G) and dispase (Roche, 04942078001). After a pre-plating step to discard contaminant cells, such as fibroblasts, myoblasts were cultured on a Matrigel-coated dish (Corning, 356231) and induced to differentiate into myotubes for 2-3 days, or for the indicated time, in differentiation medium [Iscove's modified Dulbecco's medium (Gibco, 21980-032)+2% horse serum (Gibco, 16050-122)+1% penicillin-streptomycin (Gibco, 15140-122)]. For staining myotubes, an anti-MHC antibody was used (Developmental Studies Hybridoma Bank, MF-20). For staining membranes accessible to the culture media, myotubes were incubated on ice for 10 min with FM4-64 (5 µg/ml; Thermo Fisher Scientific) in Ca²^+^ free Hank's balanced salt solution before live imaging using an SP8 inverted confocal microscope (Leica).

### Histology on newborns

Day 18.5 fetuses were skinned, fixed in Bouin's fluid for at least 1 week, decalcified in rapid decalcifier DC3 (Labonord) for 24 h with two changes, washed 24 h with three changes in 96% ethanol, dehydrated in absolute ethanol for 1 day with three changes, cleared in Histolemon for 1 day with three changes then embedded in paraplast (4 days with four changes). Serial 7 µm sections were deparaffinized with Histosol (Shandon) then stained with either Groat's hematoxylin followed by Mallory's trichrome or HE according to standard protocols.

### Histological and immunofluorescence analysis of skeletal muscle

Muscles and other tissues were frozen in nitrogen-cooled isopentane and liquid nitrogen for histological and immunoblot assays, respectively. Longitudinal and transverse 8 µm cryosections of mouse skeletal muscles were prepared, fixed and stained with antibodies against DHPRα 1 (1:100), RYR1 (1:200), α-actinin (1:1000), DNM2-R2680 (1:200), MTM1-R2827 (1:200), pan-isoform BIN1-C99D (1:50) and BIN1-R2444 (1:100) antibodies. Nuclei were detected by co-staining with Hoechst (Sigma-Aldrich) for 10 min. Samples were viewed using a TCS SP5 laser scanning confocal microscope (Leica). Air-dried transverse sections were fixed and stained with H&E, SDH, NADH-TR or Sirius Red/Fast Green staining and image acquisition performed with a slide scanner NanoZoomer 2 HT equipped with the fluorescence module L11600-21 (Hamamatsu Photonics) or a DMRXA2 microscope (Leica). Cross-sectional area (CSA) was analyzed in H&E sections from TA skeletal muscle using FIJI image analysis software. CSA (μm^2^) was calculated (>500 fibers per mouse) from 4 to 7 mice per group. The percentage of TA muscle fibers with centralized or internalized nuclei was counted in >500 fibers from 4 to 6 mice using the cell counter plug-in in ImageJ image analysis software.

### Immunofluorescence on isolated muscle fibers

Dissected muscles were fixed in 4% paraformaldehyde for 30 min at room temperature and then incubated in PBS supplemented with 0.1 M glucose for 30 min at room temperature, and afterwards in PBS supplemented with 30% sucrose at 4°C overnight. Muscles were then frozen at −80°C. Thawed entire muscles were dissected in PBS to isolate fibers. Fibers were permeabilized in PBS supplemented with 50 mM NH4Cl and 0.5% Triton X-100 for 30 min at room temperature, and then saturated in PBS supplemented with 50 mM NH_4_Cl, 0.5% donkey serum and 0.1% Triton X-100 for 1 h at room temperature. Immunofluorescence was performed at 4°C overnight using MTM1 (1:200; R2827; homemade), BIN1 (1:50, C99D), DHPR (1:150, Abcam) and RYR1 (1:150, Sigma-Aldrich) antibodies. Fibers were then incubated with donkey anti-mouse or donkey anti-rabbit secondary antibodies (Alexa Fluor 488/594, Life Technologies) diluted 1:250.

### Light microscopy

All microscopy was performed at the IGBMC Imaging Centre. All samples for microscopy were mounted in Fluorsave reagent (Merck) and viewed at room temperature. Light microscopy was performed using a fluorescence microscope (Leica, DM4000) fitted with a color charged-coupled device camera (Coolsnap cf colour, Photometrics). Confocal microscopy was performed using a confocal laser scanning microscope (TCS SP2 or SP5; Leica). ImageJ and FIJI analysis software were used for image analysis (Schneider et al., 2012).

### Transmission electron microscopy

Mice were anesthetized by intraperitoneal injection of 10 μl/g of ketamine (20 mg/ml; Virbac) and xylazine (0.4%, Rompun; Bayer) and euthanized. Muscle biopsy specimens from hindlimbs were fixed with 2.5% glutaraldehyde in 0.1 M cacodylate buffer (pH 7.2) and processed as described previously (Amoasii et al., 2012). For T-tubule analysis, potassium ferrocyanide staining was performed as described previously (Al-Qusairi et al., 2009).

### Immunogold staining

Deeply anaesthetized mice were transcardially perfused with 4% formaldehyde and 0.1 M phosphate buffer. Dissected TA were further postfixed in 4% formaldehyde and 100 µm longitudinal sections were cut using a vibratome. A standard free-floating immunocytochemical procedure was followed, using 0.1 M saline phosphate buffer as a diluent and rinsing liquids. After pre-incubation in 5% normal goat serum and 5% bovine serum albumin, sections were incubated overnight at 4°C in 1:500 anti-BIN1-2405 antibody. A further 4 h incubation with ultra-small gold conjugate of goat anti-mouse IgG (1:20; Aurion) was followed by extensive washings and 10 min postfixation in 2% glutaraldehyde, and 0.70 nm gold beads were then silver enhanced (HQ silver; Nanoprobes). After 15 min post-fixation in 1% OsO_4_, sections were dehydrated in graded acetone and finally embedded in Epon resin. Ultrathin sections were examined with a Philips CM120 electron microscope, operated at 80 kV and imaged with a SIS Morada digital camera.

### RT-PCR and qRT-PCR

Total RNA was purified from hindlimb, TA and quadriceps muscles using TRIzol reagent (Invitrogen) according to the manufacturer's instructions. cDNA was synthesized from 1–2 μg of total RNA using SuperScript II reverse transcriptase (Invitrogen) and random hexamers. qPCR amplification of cDNA was performed on a LightCycler 480 instrument (Roche). Gene expression was considered as dysregulated when fold change was higher than 1.3 and *P*≤0.05. The following primers were used:

Bin1 exon7 F, 5′-ACTATGAGTCTCTTCAAACCGCC-3′; Bin1 exon7 R, 5′-TCCACGTTCATCTCCTCGAACACC-3′; Bin1 exon9 F, 5′-TCAACACGTTCCAGAGCATC-3′; Bin1 exon19 R, 5′-GTGTAATCATGCTGGGCTTG-3′; *Bin1* exon18-19 F, 5′-CATGTTCAAGGTTCAAG-3′; *Bin1* exon20 R, 5′-TGATTCCAGTCGCTCTCCTT-3′; *Casq* 2 F, 5′-GCCCAACGTCATCCCTAACA-3′; *Casq* 2 R, 5′-GGGTCACTCTTCTCCGCAAA-3′; *Tnnt* 2 F, 5′-GCCATCGACCACCTGAATGA-3′; *Tnnt* 2 R, 5′-GCTGCTTGAACTTTTCCTGC-3′; *Jp* 2 F, 5′-ACGGAGGAACCTATCAAGGC-3′; *Jp* 2 R, 5′-CTTGAGAAAGCTCAAGCGGC-3′; *eMhc* F, 5′-AGATGGAAGTGTTTGGCATA-3′, *eMhc* R, 5′-GGCATACACGTCCTCTGGCT-3′; *Gapdh* F, 5′-TTGTGATGGGTGTGAACCAC-3′; *Gapdh* R, 5′-TTCAGCTCTGGGATGACCTT-3′.

### Western blotting

Muscle was homogenized using an Ultra-Turrax (IKA-WERKE) homogenizer in 50 mM Tris, 10% glycerol, 50 mM KCl, 0.1% SDS, 2% Triton X-100 and a set of protease inhibitors [1 mM EDTA, 10 mM NaF, 1 mM Na_3_VO_4_, 1 mM phenylmethylsulphonyl fluoride, 1 µM pepstatin and 10 µM leupeptin]. The homogenate was kept at 4°C for 2 h and clarified by centrifugation at 9400 ***g*** for 10 min. Protein concentration of the supernatant fraction was quantified using a Bio-Rad Protein Assay and lysates were analyzed by SDS-PAGE, and western blotting on a nitrocellulose membrane. Primary antibodies used were DNM2-R2680 (1:500), DNM2-R2865 (1:500), BIN1-R2405 (1:5000), BIN1-R2444 (1:500), GAPDH (1:10,000) and DNA polymerase (1:1000). Secondary antibodies were anti-rabbit HRP or anti-mouse HRP (1:10,000). Western blot films were scanned and band intensities were determined using ImageJ software. Densitometry values were standardized to corresponding total GAPDH values and expressed as a fold difference relative to the listed control (*n*=3-5 mice per group).

### Electrophysiology and fluorescence measurements in isolated muscle fibers

Single fibers were isolated from the flexor digitorum brevis (FDB) and interosseus muscles following previously described procedures (Jacquemond, 1997). Comparisons were always made between groups of fibers issued from the same muscle type. Experiments were carried out on muscle fibres isolated from the muscles of both hindlimbs from three wild-type and 3 *Bin1*ex11^−/−^ mice.

The silicone voltage-clamp technique (Jacquemond, 1997; Lefebvre et al., 2014) was used with an RK-400 patch-clamp amplifier (BioLogic) in combination with an analog-digital converter (Digidata 1440A, Axon Instruments) controlled by pClamp 9 software (Axon Instruments). Voltage-clamp was performed with a micropipette filled with a solution mimicking the ionic composition of the cytosol and also containing either a high concentration of EGTA and the fluorescent Ca^2+^-sensitive probe rhod-2 (see the full composition below), or the contraction blocking agent N-benzyl-p-toluene sulphonamide (50 μM) and the fluorescent Ca^2+^-sensitive probe fluo-4 FF (100 μM). Intracellular equilibration of the solution was allowed for 30 min. For calcium current analysis, the linear leak component was removed as described previously (Kutchukian et al., 2017). The voltage dependence of the peak current was fitted with the following equation: *I*(*V*)=*G_max_*(*V*-*V_rev_*)/[1+exp(*V_0.5_*-*V*)/*k*] with *I*(*V*) the peak current density at the command voltage, *V*, *G_max_* the maximum conductance, *V_rev_* the apparent reversal potential, *V_0.5_* the half-activation potential and *k* the steepness factor.

Confocal imaging was conducted with a Zeiss LSM 5 Exciter microscope equipped with a 63× oil immersion objective (numerical aperture 1.4). For detection of rhod-2 and fluo-4 FF fluorescence, excitation was from the 543 nm line of a HeNe laser and from the 488 nm line of an Argon laser, respectively, and fluorescence was collected above 560 nm and above 505 nm, respectively. Rhod-2 and fluo-4 FF Ca^2+^ transients were imaged using the line-scan mode (*x*,*t*) of the system. Rhod-2 and fluo-4 FF fluorescence changes were expressed as F/F_0_, where F_0_ is the baseline fluorescence. Quantification of the spatial heterogeneity of Ca^2+^ release activation and of the absolute Ca^2+^ release flux underlying the rhod-2 Ca^2+^ transients were performed as described previously (Kutchukian et al., 2017). In each fiber, the voltage dependence of the peak rate of Ca^2+^ release was fitted with a Boltzmann function. For imaging the T-tubule network, FDB muscle fibers were incubated for 30 min in the presence of 10 µM di-8-anepps in Tyrode’s solution. For di-8-anepps, fluorescence was collected above 505 nm with 488 nm excitation. The T-tubule density from the di-8-anepps fluorescence was estimated as described previously (Kutchukian et al., 2017).

Tyrode's solution contained (in mM): 140 NaCl, 5 KCl, 2.5 CaCl_2_, 2 MgCl_2_ and 10 HEPES. The extracellular solution used for voltage-clamp contained (in mM): 140 TEA-methanesulfonate, 2.5 CaCl_2_, 2 MgCl_2_, 1 4-aminopyridine, 10 HEPES and 0.002 tetrodotoxin. The pipette solution contained (in mM): 120 K-glutamate, 5 Na_2_-ATP, 5 Na_2_-phosphocreatine, 5.5 MgCl_2_, 15 EGTA, 6 CaCl_2_, 0.1 rhod-2, 5 glucose and 5 HEPES. All solutions were adjusted to pH 7.2.

### Statistical analysis

As the compared groups came from the same background, statistical analyses were performed using a two-tailed unpaired Student's *t*-test, unless stated otherwise. The number of animals and experiments were based on previous reported data using the same experimental tests on wild-type and myopathic mice. No samples or animals were excluded from the analyses. *P*-values of <0.05 were considered significant.

## Supplementary Material

Supplementary information
